# Pushing the Boundaries of Ampullectomy for Benign Ampullary Tumors: 25-Year Outcomes of Surgical Ampullary Resection Associated with Duodenectomy or Biliary Resection

**DOI:** 10.3390/jcm13237220

**Published:** 2024-11-27

**Authors:** Maria Sorribas, Thiago Carnaval, Luis Secanella, Núria Peláez, Silvia Salord, Joan B. Gornals, David Leiva, Teresa Serrano, Joan Fabregat, Juli Busquets

**Affiliations:** 1 Digestive and General Surgery Department, Bellvitge University Hospital, L’Hospitalet de Llobregat, 08907 Barcelona, Spain; sorribasgrifell@gmail.com (M.S.); lsecanella@bellvitgehospital.cat (L.S.); npelaez@bellvitgehospital.cat (N.P.); fabregatj139@gmail.com (J.F.); 2 Research Group of Hepato-Biliary and Pancreatic Diseases, Institut d’Investigació Biomèdica de Bellvitge—IDIBELL, University of Barcelona, L’Hospitalet de Llobregat, 08907 Barcelona, Spain; 3 Oncology Data Analytics Program (ODAP), Catalan Institute of Oncology (ICO), L’Hospitalet de Llobregat, 08908 Barcelona, Spain; thcarnac7@alumnes.ub.edu; 4 Gastroenterology Department, Bellvitge University Hospital, L’Hospitalet de Llobregat, 08907 Barcelona, Spain; ssalord@bellvitgehospital.cat (S.S.); jgornals@bellvitgehospital.cat (J.B.G.); 5 Radiology Department, Bellvitge University Hospital, L’Hospitalet de Llobregat, 08907 Barcelona, Spain; dleiva@bellvitgehospital.cat; 6 Pathology Department, Bellvitge University Hospital, L’Hospitalet de Llobregat, 08907 Barcelona, Spain; tserrano@bellvitgehospital.cat; 7 Departament de Ciències Clíniques, Facultat de Medicina i Ciències de la Salut, Universitat de Barcelona (UB), C. Casanova, 143, 08036 Barcelona, Spain

**Keywords:** ampullary tumor, ampullectomy, transduodenal ampullectomy, pancreaticoduodenectomy

## Abstract

**Background:** Surgical resection for ampullary lesions lacks clear guidelines. Pancreaticoduodenectomy (PD) is the standard treatment for malignant ampullary tumors but is often excessive for ampullary adenomas (AAs) due to its high morbidity and mortality. Transduodenal ampullectomy (TDA) is generally reserved for small benign lesions where endoscopic treatment fails, but its role in early ampullary cancers is debatable. This study presents our 25-year outcomes with TDA for benign ampullary tumors. **Methods:** We conducted a retrospective cohort study with prospectively collected data from patients with benign ampullary lesions who underwent TDA between January 1996 and November 2023. Primary outcomes were the 30-day overall and severe (Clavien–Dindo ≥ IIIa) morbidity rates and the 90-day mortality rate. Categoric variables were presented as absolute and relative frequencies, and quantitative variables were presented as means (standard deviation, SD) or medians (range or interquartile range, IQR). **Results:** Fifty-three patients (29 male; mean [SD] age 62.5 [14.6] years) underwent TDA. The 30-day morbidity rate was 32.1% (17/53 patients), with five (9.4%) cases being severe. The 90-day mortality rate was 1.9%. Definitive histopathology identified 38 (71.7%) AAs and five (9.4%) infiltrating ampullary adenocarcinomas, two (40.0%) of which required subsequent PD. Six (11.3%) patients experienced recurrence. Overall, nine (16.9%) patients died. **Conclusions:** TDA is a safe and effective technique with acceptable morbidity for non-infiltrating lesions, especially in patients with poor clinical status. Choosing between TDA and PD depends on tumor size, dysplasia grade, and institutional expertise. Lifelong endoscopic surveillance post-TDA is essential for timely recurrence detection.

## 1. Introduction

Benign neoplasms of the ampulla of Vater are rare, and ampullary adenomas (AAs) are the most common type, with a reported prevalence of 0.04–0.12% based on historical autopsy data [1,2,3,4,5,6,7]. However, advancements in endoscopic surveillance and computed tomography (CT) have increased detection rates in recent years [3]. Although AAs are often asymptomatic, they may cause recurrent pancreaticobiliary complications, such as jaundice, cholangitis, pancreatitis, and bleeding [6]. More importantly, AAs are recognized as pre-cancerous lesions following the same adenoma–carcinoma sequence observed in other gastrointestinal adenomas, underscoring the need for precise diagnosis and appropriate management [8,9].

Differentiating AAs from ampullary adenocarcinomas (AACs) remains challenging, even with comprehensive preoperative assessments, as AAs may harbor high-grade dysplasia (HGD) areas that can be unintentionally overlooked, resulting in high false-negative rates [6,10]. Consequently, treatment strategies have evolved to balance completeness of resection with morbidity and mortality risks. Current approaches include endoscopic papillectomy (EP), transduodenal ampullectomy (TDA), and pancreaticoduodenectomy (PD). EP is often preferred for small (<1 cm) lesions confined to the papilla and not involving the common bile or pancreatic ducts, given its low morbidity and mortality rates [11,12]. However, limitations in current diagnostic techniques carry a risk of missing sites of malignant transformation, which could result in incomplete resection with EP. For patients not eligible for EP due to specific factors such as tumor size, location, or patient anatomy, surgical options may be necessary. PD, the standard approach for malignant ampullary tumors, carries considerable postoperative morbidity (33–52%) and mortality (2–5%), even in high-volume centers [13], which might render it an overtreatment for AAs [12]. TDA offers a less invasive alternative, particularly for small, benign lesions or after failed endoscopic treatment, though its role in early ampullary cancers remains debatable [12].

Indications for surgical ampullary resection are still evolving, with therapeutic choices often depending on institutional expertise and available resources [14,15,16]. Over the past 25 years, our center has used TDA to preserve pancreatic parenchyma while managing different ampullary lesions. This technique has been applied for both non-adenomatous (e.g., gastrointestinal stromal tumors, intraductal mucinous papillary neoplasm of the biliary duct, and ampullary neuroendocrine tumors) and adenomatous lesions, including cases with bile duct or duodenal involvement and patients with poor clinical status (i.e., cirrhosis and short bowel syndrome) for whom PD would pose an unacceptable risk of morbidity. This study aims to describe our experience and outcomes with TDA in benign ampullary lesions, offering insights to support clinical decision-making.

## 2. Materials and Methods

### 2.1. Study Design, Population, and Setting

We conducted a single-center retrospective cohort study with prospectively collected data from patients diagnosed with benign ampullary lesions who underwent TDA and were followed at our tertiary hospital in the southern metropolitan area of Barcelona, between January 1996 and November 2023. This study received Institutional Review Board approval from the Bellvitge University Hospital Ethics Committee on 21 December 2023 (PR303/23) and was granted a waiver of informed consent due to its retrospective nature and use of anonymized clinical data. This study adhered to the Declaration of Helsinki, Good Clinical Practice guidelines, and applicable Spanish and European regulations. Confidentiality was ensured according to the current Spanish (LOPD 3/2018) and European (EU Regulation 2016/679 of the European Parliament and Council of 27 April 2016) data protection legislations.

This manuscript complies with the Strengthening the Reporting of Observational Studies in Epidemiology (STROBE) guidelines [17].

### 2.2. Eligibility Criteria

We included adult patients (≥18 years of age), of both sexes, diagnosed with an ampullary lesion requiring surgical ampullectomy. We excluded those with evidence (in the preoperative frozen section biopsy) of an infiltrating AAC.

### 2.3. Study Procedures

#### 2.3.1. Diagnostic Work-Up and Therapeutic Approach

Patients with obstructive jaundice and no detectable tumor on the abdominal CT scan underwent endoscopic examination with visual assessment of the major papilla, during which biopsy samples were collected. Preoperative bile duct drainage was performed in patients with hyperbilirubinemia (bilirubin > 300 µmol/L) or hypoalbuminemia (albumin < 35 g/dL). All patients referred from other centers for benign ampullary lesions were reassessed comprehensively, and their preoperative biopsies were reviewed by our center’s expert pathologist. Ampullectomy was only performed after confirming a benign ampullary lesion.

Since 2008, we have routinely used preoperative magnetic resonance cholangiography to improve anatomical assessment of the biliopancreatic junction and rule out conditions such as pancreas *divisum*. From 2011, EP was implemented for small (<1 cm) lesions with benign endoscopic appearance and no evidence of endobiliary growth on endoscopic ultrasound (EUS), aiming to refine non-invasive management.

#### 2.3.2. Surgical Technique

**Figure 1:**
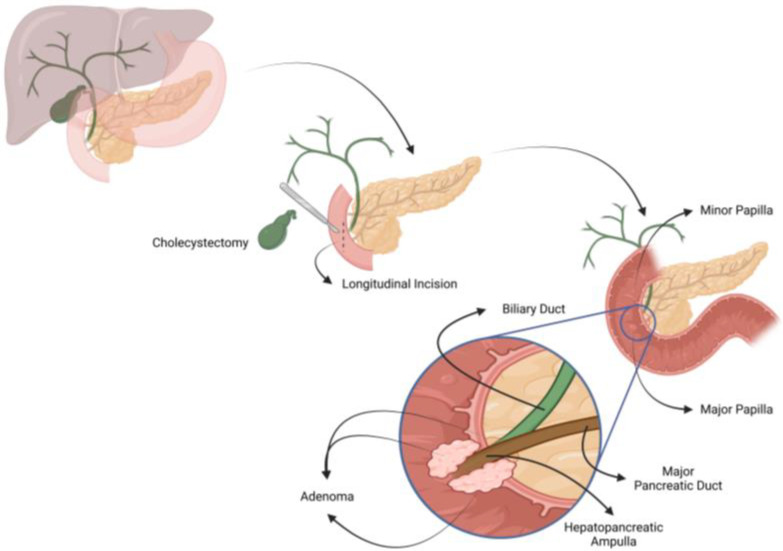
Transduodenal ampullectomy. Created in BioRender. Carnaval, T. (2023) BioRender.com/t97f441 (accessed on 14 October 2024).

A right subcostal laparotomy incision was made, followed by cavity exploration, cholecystectomy, and bile duct stenting using a Nelaton catheter. A wide Kocher maneuver was performed to expose the pancreatic head and ampulla of Vater. After identifying the ampulla and the tumor, a five-centimeter longitudinal duodenotomy, centered on the ampulla, was created along the duodenal free edge (Figure 1). Disease-free mucosa around the tumor was incised, followed by submucosa incision and careful sectioning of healthy bile and pancreatic ducts. Both ducts were marked with 5-0 monofilament sutures. Complete tumor resection was confirmed through frozen section biopsy.

#### 2.3.3. Frozen Section Evaluation

All resected ampullary tumors underwent perioperative frozen section histopathological evaluation to assess both tumor characteristics and margins. If an infiltrating AAC was detected, the procedure was immediately converted to a cephalic PD during the same operation, and these cases were subsequently excluded from this study. Margins were extended when HGD was present. In case of evidencing dysplasia at the common bile duct margin, resection was extended distally along the bile duct until clear margins were achieved. Patients requiring full bile duct resection underwent hepaticojejunostomy.

Definitive postoperative histopathological assessment was conducted for all resected samples. If an infiltrating AAC was identified, an individualized treatment plan was developed based on the patient’s nutritional and physical status.

#### 2.3.4. Intestinal Reconstruction

Choledochoduodenostomy and pancreaticoduodenostomy were performed using a single-layer circumferential suture technique. Duodenorrhaphy was performed longitudinally (Video S1). For cases of pancreas *divisum* with the main duct draining to the minor papilla, only choledochoduodenostomy was performed. A passive drainage system was placed near the duodenostomy site, and a suction nasogastric catheter was maintained for the first 12 h following surgery.

### 2.4. Outcomes

The primary outcomes were the 30-day morbidity rates, both overall and severe (using the Clavien–Dindo classification, grade ≥ IIIa), and the 90-day mortality rate. Secondary outcomes included the following: (i) the median (range) hospital stay; (ii) the number (percentage) of reinterventions; (iii) the number (percentage) of hospital readmissions; (iv) the number (percentage) of long-term disease recurrences; and (v) the long-term mortality rate.

### 2.5. Data Sources

This retrospective study used a prospectively maintained database of patients initially diagnosed with benign ampullary tumors (based on biopsy conducted during the endoscopic assessment of the major papilla) who underwent TDA between January 1996 and November 2023. Baseline and sociodemographic characteristics, perioperative details, and short- and long-term postoperative outcomes were retrieved.

The database included anonymized information, accessible only to the principal investigator and authorized study team members, and confidentiality was ensured per Spanish (LOPD 3/2018) and European (EU Regulation 2016/679 of the European Parliament and Council of 27 April 2016) data protection laws. All data were dissociated at entry, assigning a unique code to each patient.

### 2.6. Sample Size and Statistical Analysis

Due to the descriptive nature of this study, no formal sample size calculation was performed. Descriptive analyses were performed for all study variables, with categoric variables reported as absolute and relative frequencies and quantitative variables as means (standard deviation, SD) or medians (range or interquartile range, IQR), as appropriate. All statistical analyses were performed with SPSS Statistics^®^ for Windows^®^, version 18.0 (SPSS Inc., Chicago, IL, USA).

## 3. Results

### 3.1. Baseline Characteristics and Short-Term Results

A total of 53 patients underwent TDA following an initial diagnosis of benign ampullary tumors (Table 1). The mean (SD) age was 62.5 (14.6) years, with 29 (54.7%) male and 24 (45.3%) female patients. The mean (SD) operative time was 257.0 (54.0) minutes. Among these patients, three (5.7%) had a prior diagnosis of cirrhosis, five (9.4%) required hepaticojejunostomy due to distal bile duct involvement, and three (5.7%) did not undergo pancreaticoduodenostomy due to pancreas *divisum*.

In the first 30 days post-surgery, complications were observed in 17 patients (Table 2), yielding a morbidity rate of 32.1%. Severe complications (Clavien-Dindo ≥ IIIa) occurred in five (9.4%) patients, including two (3.8%) cases of severe acute pancreatitis, two (3.8%) cases of type C pancreatic fistulae, and one (1.9%) case of unintended intestinal perforation. Only one patient died during the first 90 days after TDA, yielding a 1.9% mortality rate.

The median (range) hospital stay was 8 (6–50) days. Only three (5.7%) patients required surgical reintervention: two (3.8%) due to severe acute pancreatitis and one (1.9%) for intestinal perforation. Additionally, five (9.4%) patients were readmitted, all because of an intra-abdominal abscess.

### 3.2. Long-Term Results

**Figure 2:**
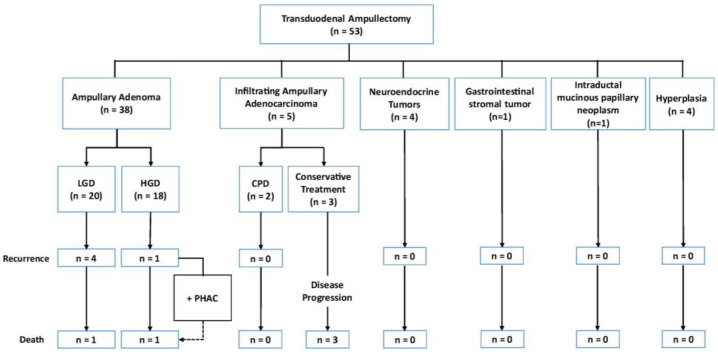
Results from the definitive histopathological assessment. CPD: cephalic pancreaticoduodenectomy; HGD: high-grade dysplasia; LGD: low-grade dysplasia; PHAC: pancreatic head adenocarcinoma.

All patients underwent a preoperative endoscopic study with assessment of the major papilla and biopsy, with no initial signs of malignancy. However, the postoperative definitive histopathological assessment (Figure 2) showed that 38 of the 53 (71.7%) tumors were AAs, 5 (9.4%) were infiltrating AACs, 1 (1.9%) was a gastrointestinal stromal tumor (GIST), 1 (1.9%) was an intraductal mucinous papillary neoplasm of the bile duct, 4 (7.6%) were ampullary neuroendocrine tumors, and 4 (7.6%) showed ampullary hyperplasia without neoplasm (a benign condition that might mimic adenomas or other lesions and lead to diagnostic uncertainty during imaging and endoscopy). Among those with infiltrating AACs, two (40.0%) patients underwent subsequent cephalic PD, while the remaining three (60.0%) were managed conservatively due to comorbidities that contraindicated more aggressive approaches.

All patients underwent periodic visual assessment of the major papilla and biopsies postoperatively. The median (IQR) follow-up period was 51.6 (17.2–140.7) months. Six (11.3%) patients experienced disease recurrence, including one case of early recurrence (within the first year after surgery) and five cases of late recurrence (≥3 years post-TDA). One patient with late recurrence also developed a de novo pancreatic head adenocarcinoma eight years after TDA and subsequently underwent cephalic PD.

In total, nine patients died over the follow-up period, yielding a 16.9% long-term mortality rate. Among these, four (44.4%) deaths were due to causes unrelated to the TDA procedure or the ampullary lesion. Table 3 summarizes the causes of death.

### 3.3. Expanded Indications for Transduodenal Ampullectomy

In five (9.4%) patients, TDA was performed with bile duct resection and reconstruction using both pancreaticoduodenal and bilioenteric anastomoses. Of these, three (60.0%) involved adenomatous ampullary lesions with endobiliary growth, one (20.0%) involved an intraductal mucinous papillary neoplasm of the bile duct, and one (20.0%) involved an AAC identified intraoperatively, where PD was deemed unfeasible due to advanced age and comorbidities.

One (1.9%) patient with an adenomatous ampullar lesion extending to the third duodenal portion underwent TDA with D3-duodenectomy. Choledochoduodenal and pancreaticoduodenal ductomucosal sutures were performed in a crown pattern, along with an infrapapillary duodeno–duodenal suture (Video S1). For a lesion affecting nearly the entire circumference, TDA was accompanied by complete circumferential duodenal mucosectomy.

TDA was also performed in one (1.9%) patient with short bowel syndrome due to multiple previous abdominal surgeries aiming to preserve as much intestinal tissue as possible, given the high-grade AA diagnosis.

Lastly, in one (1.9%) cirrhotic patient with previous episodes of ascitic-edematous decompensation, surgical TDA was chosen following the diagnosis of a GIST.

## 4. Discussion

Determining the optimal therapeutic approach for ampullary lesion remains challenging due to the limited observational evidence available, leading to reliance on each center’s expertise [18,19,20,21,22,23]. While EP is a valid option, it carries a considerable risk of incomplete resection, potentially requiring additional interventions. In turn, surgical options like ampullectomy and PD may achieve higher complete resection rates but are often associated with increased morbidity and mortality, particularly in patients with comorbidities or advanced age. This article describes our 25-year experience with TDA for benign ampullary lesions, highlighting favorable short- and long-term outcomes and offering insights into a potential alternative within this complex therapeutic landscape.

The indications for TDA remain under debate, with AAs being the least controversial. TDA plays an intermediate role between EP and PD in terms of extensiveness and associated morbidity [8]. Smaller tumors are often treated with EP, whereas larger tumors unsuitable for EP may be managed more effectively with TDA [8,12,15,18,22,24]. The European Society of Gastrointestinal Endoscopy (ESGE) recommends EP for patients with AAs up to 20–30 mm in diameter without intraductal extension, advising submucosal injection before resecting laterally spreading duodenal ampullary tumors to enable safe and effective endoscopic mucosal resection (EMR) [25]. Alternatively, some authors reported using endoscopic submucosal dissection (ESD) in cases of recurrent, laterally spreading papillary adenomas < 2 cm, successfully achieving en bloc resection [26]. Others combined miniprobe ultrasonography guidance and ESD and successfully achieved en bloc resection of HGD AAs > 2 cm with no evidence of deep tissue invasion [27]. Anecdotally, a case report described the successful use of a combined ESD-EMR procedure for a large (4 cm) ampullary lesion [28]. However, given the technical complexity and risk profile, ESD should only be performed by experienced hands [26,28].

Accurate diagnosis is crucial for guiding treatment decisions and selecting appropriate therapeutic strategies. EUS is highly effective in detecting AAs, offering good accuracy for T-staging—particularly in identifying submucosal and muscularis invasion—while matching the performance of endoscopic retrograde cholangiopancreatography (ERCP) in evaluating the intraductal extension of AAs [29]. EUS findings play a significant role in therapeutic decision-making, showing potential in up- or downstaging AAs and directly influencing whether endoscopic or surgical resection is pursued [30,31].

Comparatively, endoscopic biopsy alone may yield up to 60% false negatives and has only about 45% accuracy in identifying infiltrating AACs [32]. However, when combined with perioperative frozen section analysis, diagnostic accuracy can improve to nearly 100% [19]. Although perioperative frozen section evaluation is a widely used and accepted practice when resecting AAs, discrepancies with the final histopathological reports are relatively common [33] and are influenced by factors such as sample quality, processing, and pathologist expertise. In our cohort, 22% of frozen section results differed from the definitive histopathological assessments; however, only 4% involved a misdiagnosis of AAC in the frozen section. Thus, while perioperative frozen sections provide valuable intraoperative guidance, cautious interpretation and subsequent confirmation with definitive histopathological assessment remain essential.

Furthermore, relying single-handedly on tumor size could be misleading. While N0 and M0 lesions are prerequisites for TDA, considerations such as tumor size, intraductal extension, T-stage, and histological grade remain unsettled. The recommended threshold for TDA is often a maximum tumor size of 2–3 cm [8,34,35], though tumors ≥ 1 cm may carry an increased risk of lymph node metastasis [13]. In our cohort, 17 patients with HGD AAs underwent TDA, with only one (5.9%) case of recurrence, in which the patient developed a de novo pancreatic head adenocarcinoma several years later, despite the thorough follow-up. Interestingly, patients with low-grade dysplasia had a higher recurrence rate post-TDA. Given that HGD/pTis lesions may still involve lymph node metastasis (despite having intact lamina propria), we support TDA as a safe, less aggressive option for AAs, regardless of dysplasia grade. However, PD should be the preferred alternative for lesions ≥ pT1 in patients fit for surgery, due to the risk of lymph node metastasis.

Intraductal extensions up to 10 mm are generally acceptable for EP if the lesion is a low- or high-grade dysplasia [15]. In contrast, some authors indicate TDA for well- or moderately-differentiated pTis/pT1 tumors < 2 cm with <10 mm ductal extension and no lymph node involvement [21]. More conservative approaches advocate PD for T1 tumors in eligible patients due to a lymph node metastasis risk of up to 30% [13,36]. In our practice, TDA was performed regardless of tumor size or bile duct/duodenal involvement, and our results support this approach. Notably, infrapapillary D3 duodenectomy—previously reported by our group as a pancreatic-preserving technique for extra-duodenal tumors with duodenal invasion [37]—may also benefit selected patients; however, combining TDA with infrapapillary D3 duodenectomy for AAs with duodenal involvement has not been previously reported.

Therapeutic decisions should be tailored and decided by a multidisciplinary team. Unsurprisingly, a recent meta-analysis reported superior resection rates with surgical approaches than with endoscopic therapy, with pooled R0 rates of 76.6% for EP, 96.4% for TDA, and 98.9% for PD; however, morbidity rates were also higher (24.7%, 28.3%, and 44.7%, respectively) [38]. Notably, TDA generally requires less operative time and shorter hospital stays than PD, making it a less costly alternative [39,40]. However, we cannot draw conclusions on cost-effectiveness without a full economic analysis. Although TDA has slightly higher recurrence rates compared to PD [21,34], lifelong endoscopic surveillance after TDA remains essential to detect potential recurrences [12,41], except in cases of confirmed papillary hyperplasia. Ultimately, the choice of procedures should balance technical feasibility with the goals of complete resection and minimizing morbidity, tailoring the approach to each patient’s clinical condition, lesion characteristics, and the available expertise within the institution.

This study has limitations. Patient heterogeneity, possible selection bias, and the small sample size may limit the generalizability of our findings. Moreover, advancements in clinical management and diagnostic technology over the years may affect cohort consistency. Finally, we lacked data on patients’ quality of life, which could have provided further insight into the intervention’s impact on well-being.

## 5. Conclusions

Although TDA has a higher recurrence rate than PD, it is a safe and effective technique with acceptable morbidity, particularly suited for patients with non-infiltrating lesions and poor clinical status. Choosing between TDA and PD should consider factors such as tumor size, dysplasia grade, lesion location, and institutional expertise. Importantly, endobiliary or intraduodenal growth should not preclude TDA. Except in cases of confirmed papillary hyperplasia, lifelong endoscopic surveillance following TDA is crucial for the timely detection of recurrences in case of AAs.

## Figures and Tables

**Table 1:** Baseline and sociodemographic characteristics.

Baseline and Sociodemographic Characteristics	N = 53
**Age** (**years**)**, mean** (**SD**)	62.5 (14.6)
**Sex, n** (**%**)	
Male	29 (54.7)
Female	24 (45.3)
**ASA, n** (**%**)	
I	10 (18.9)
II	23 (43.4)
III	20 (37.7)

ASA: American Society of Anesthesiologists Classification.

**Table 2:** Postoperative morbidity.

Postoperative Morbidity	N = 53
**Infections**	
Intra-abdominal Abscess	9 (17.0)
Surgical Site Infection	9 (17.0)
Other Infections	5 (9.4)
**Acute Pancreatitis**	5 (9.4)
**Fistulae**	
Pancreatic Fistula	4 (7.6)
Biliary Fistula	2 (3.8)
Intestinal Fistula	4 (7.6)
**Bleeding**	
Intra-abdominal Bleeding	2 (3.8)
Upper Gastrointestinal Bleeding	1 (1.9)
**Blood Transfusion Requirement**	
≤48 h	2 (3.8)
>48 h	7 (13.2)
**Delayed Gastric Emptying**	3 (5.7)
**Parenteral Nutrition Requirement**	10 (18.9)

**Table 3:** Long-term mortality.

Long-Term Mortality	N = 9
Postoperative death (≤90 days)	1 (11.1)
Recurrent disease + de novo pancreatic head adenocarcinoma	1 (11.1)
Non-intervened infiltrating ampullary adenocarcinoma	3 (33.3)
Unrelated with TDA/ampullary lesion	4 (44.4)

TDA: Transduodenal Ampullectomy.

## Data Availability

Data sets used and/or analyzed during this study are available from the corresponding author upon reasonable request.

## Supplementary Materials

The following supporting information can be downloaded at https://www.mdpi.com/article/10.3390/jcm13237220/s1: Video S1: Transduodenal Ampullectomy.
